# Lipid bilayer-atomic force microscopy combined platform records simultaneous electrical and topological changes of the TRP channel polycystin-2 (TRPP2)

**DOI:** 10.1371/journal.pone.0202029

**Published:** 2018-08-22

**Authors:** Sumit Lal, Noelia Scarinci, Paula L. Perez, María del Rocío Cantero, Horacio F. Cantiello

**Affiliations:** 1 Nephrology Division and Electrophysiology Core, Massachusetts General Hospital, Charlestown, Massachusetts, United States of America; 2 Laboratorio de Canales Iónicos, Instituto Multidisciplinario de Salud, Tecnología y Desarrollo, IMSaTeD (UNSE-CONICET), Santiago del Estero, Argentina; 3 Harvard Medical School, Boston, Massachusetts, United States of America; Newcastle University, AUSTRALIA

## Abstract

Ion channels are transmembrane proteins that mediate ion transport across biological membranes. Ion channel function is traditionally characterized by electrical parameters acquired with techniques such as patch-clamping and reconstitution in lipid bilayer membranes (BLM) that provide relevant information such as ionic conductance, selectivity, and gating properties. High resolution structural information of ion channels however, requires independent technologies, of which atomic force microscopy (AFM) is the only one that provides topological features of single functional channel proteins in their native environments. To date practically no data exist on direct correlations between electrical features and topological parameters from functional single channel complexes. Here, we report the design and construction of a BLM reconstitution microchamber that supports the simultaneous recording of electrical currents and AFM imaging from single channel complexes. As proof-of-principle, we tested the technique on polycystin-2 (PC2, TRPP2), a TRP channel family member from which we had previously elucidated its tetrameric topology by AFM imaging, and single channel currents by the BLM technique. The experimental setup provided direct structural-functional correlates from PC2 single channel complexes that disclosed novel topological changes between the closed and open sub-conductance states of the functional channel, namely, an inverse correlation between conductance and height of the channel. Unexpectedly, we also disclosed intrinsic PC2 mechanosensitivity in response to external forces. The platform provides a suitable means of accessing topological information to correlate with ion channel electrical parameters essential to understand the physiology of these transmembrane proteins.

## Introduction

TRP channels are multifunctional sensors that control cell function in response to a wide variety of stimuli, including chemical, electrical, thermal, and mechanical signals [1, 2]. The characterization of ion channels usually entails gaining information on electrical properties such as ionic conductance, selectivity and permeation, as well as gating and modulation by means of suitable techniques such as patch clamping [3] and BLM reconstitution [4]. Structural information is also essential for the understanding of ion channel function, despite the fact that it is usually attained with much more difficulty, and by independent means [5]. Cryo-electron microscopy (EM) [6–8] and X-ray crystallography [9, 10] provide highly comprehensive, but static pictures of channel structures. Single-particle cryo-EM is particularly useful because it provides structural information of ion channels in their native aqueous environment [11, 12]. Cryo-EM structural reconstruction of TRP channels such as TRPV1 and TRPV4 for example, revealed a tetrameric topology with four-fold symmetric structure [13, 14]. Because of their diverse gating properties, hetero-oligomerization, and regulatory mechanisms, structure-function correlations of TRP channels may also provide essential information as to their emerging role as drug targets in a number of disorders ranging from pain, and inflammation, to neurological defects, and polycystic kidney disease [15–22].

AFM is a high resolution imaging technology that provides topological information of ion channel proteins [23–26]. In combination with other high-resolution techniques such as cryo-EM, AFM imaging also has offered nanometer resolution from a number of TRP channels. Sato’s group disclosed bell-shaped structures for TRPC3 and TRPM2 [27, 28]. We also used AFM imaging in solution to determine the tetrameric topology of two other TRP channels, homomeric PC2 (TRPP2), and TRPC1, as well as hetero-complexes of PC2 with TRPC1 [24]. Our study also provided electrophysiological information of identical samples reconstituted in a BLM system. To date, however, there is no direct information on how structural features correlate with electrical properties of functional ion channels in their natural environment. Direct structural-functional correlations may disclose relevant information of specific topological features associated with the electrical properties of ion channels.

Here, we report on the design and construction of a BLM platform for the simultaneous acquisition of AFM images and electrical recordings from identifiable single ion channel complexes. The combined approach provided evidence for both an inverse correlation between the channel’s height and its ionic conductance, and a yet unknown intrinsic mechanosensitivity of PC2. The technique described here opens up the possibility to study high resolution structural-functional correlations from “live” single ion channel complexes.

## Materials and methods

### Schematics of connections and assembly of the BLM-AFM microchamber

**Fig 1:**
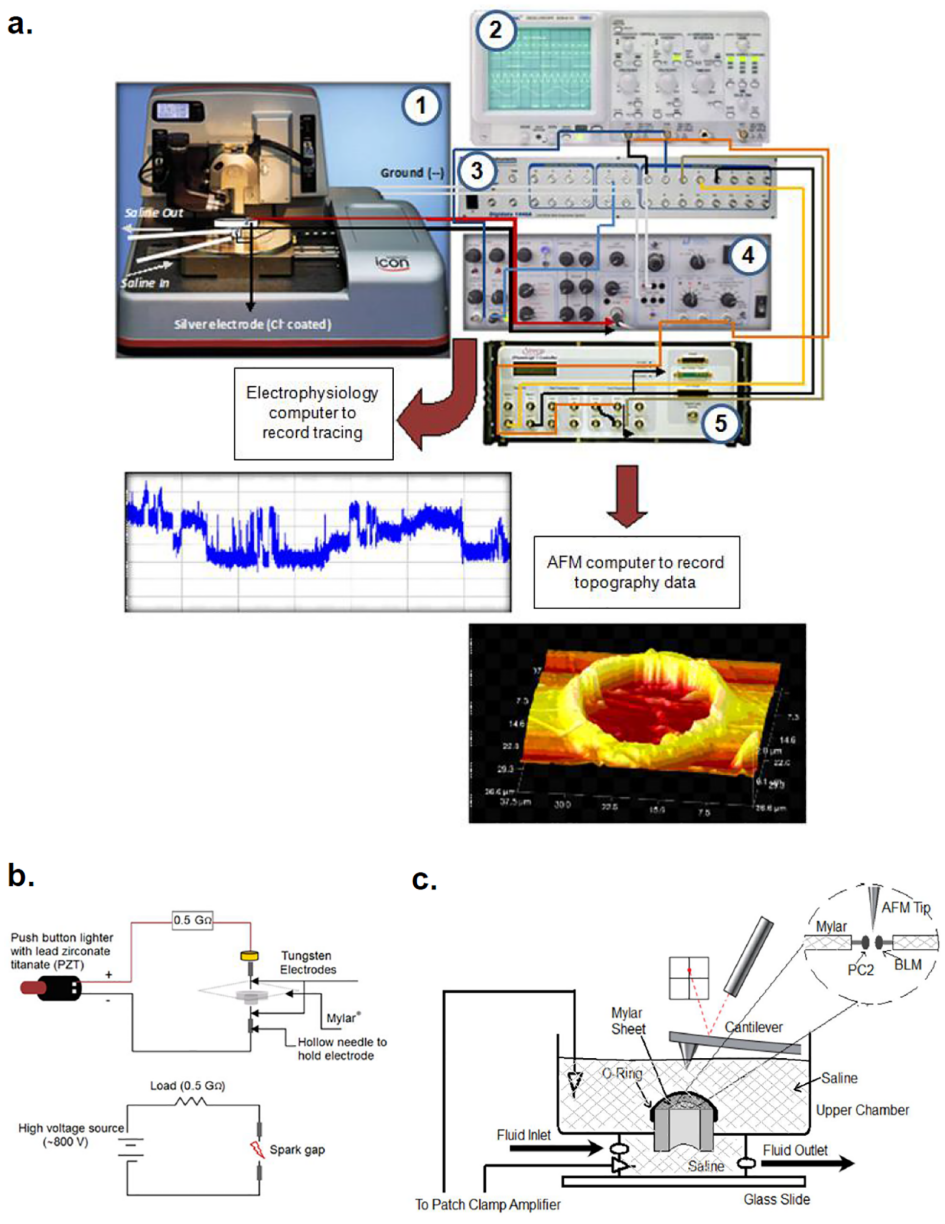
Schematics of connections and assembly of the BLM-AFM microchamber. **a.** Assembly of the system, including the AFM (1) and path clamping system, containing the patch clamp amplifier (4), connected to both a digital oscilloscope (2), and an A/D converter (3), to feed electrical signals into a PC. Signals were also inputted into an analog I/O port of the AFM controller (NanoScope V (5)), to correlate single channel currents with AFM scans (see Figs 2–5). **b.** Schematics of the hole-making, voltaic arc circuit created to melt Mylar sheets. **c.** Schematics of the BLM microchamber consisting of a nylon spacer whose top supported a stretched Mylar film containing a defined hole that separated the upper and lower hemi-chambers. Both hemi-chambers contained saline solution. The upper hemi-chamber was open to access the AFM cantilever.

The combined BLM microchamber system for the simultaneous acquisition of single channel electrophysiological and AFM data is outlined in Fig 1a–1c, with a description of the actual equipment and connections. Fig 1b shows the schematics of the hole-making circuitry and the voltaic arc connections used to melt the 3-μm-thick Mylar sheet. The home-made voltaic arc delivery system consisted of a spark generator (see below) connected in series with two 10 μm tip diameter tungsten electrodes in between which the Mylar film was placed.

### Hole-making system design

The three μm-thick Mylar film (BoPET, Biaxially-oriented polyethylene terephthalate) (a kind gift of Scott Latvalla, Steinerfilm, Inc., Williamstown, MA) was cut in square sections (1 cm^2^), soaked overnight in 100% ethanol and dried before use. Holes were made by melting a defined circular area with a home-made voltaic arc delivery system (Fig 1b), that consisted of two 10 μm tip diameter tungsten electrodes (HEKA Elektronik, Gmbh, Lambrecht, Germany), the top one mounted on a vertical manipulator to approach the tip onto the stretched surface. Tip electrodes were connected in series with a spark generator (Piezo Spark Generator, Model number 03100), via a high precision parallel circuit of two 1 GΩ resistors, ELTECH#104; Silvertech Ltd., Horsham, UK). The sparking system consistently produced 10–20 μm diameter holes in the Mylar film. The processed film was then mounted on the opening 4.5 mm (diameter) on the top of the hemi-chamber, and secured in place with a standard rubber O-ring placed at the center of an open Petri Dish. The size and shape of the hole was assessed under DIC with an Olympus IX71 inverted microscope (x60) later confirmed by AFM scanning.

### Construction of support chamber

The horizontal BLM chamber consisted of two hemi-chambers (Fig 1c). The bottom hemi-chamber was assembled from a nylon spacer (OD = 0.500", ID = 0.140", L = 0.250") that was glued to a glass microscope slide with hot melt adhesive glue. Two 1/16" holes were drilled to accommodate butterfly catheter tubing for filling and flushing of saline. One 3/32" hole was drilled to accommodate a rubber seal from Warner Instruments (Hamden, CT), through which a chloride-plated silver wire was passed through a 23 gauge hypodermic needle. A second, smaller spacer was also glued on top of the first one, to build a septum to support the interface piece containing a stretched Mylar film onto the external spacer. A small Petri dish fitted with a central O-ring, was used to create the upper chamber that was mounted onto the external spacer. The open upper chamber was accessible for lipid painting from the outside. Silver wire electrodes were electroplated by exposure to HCl (0.1 N), through a circuit connected in series to a 9 V battery. Electrodes were placed on either side of the Mylar film, respectively, and connected to the high impedance headstage (10 GΩ) of the patch clamping amplifier (PC501A, Warner Instruments, Hamden, CT).

### *In vitro* transcription-translation of PC2 and proteoliposome preparation

**Fig 2:**
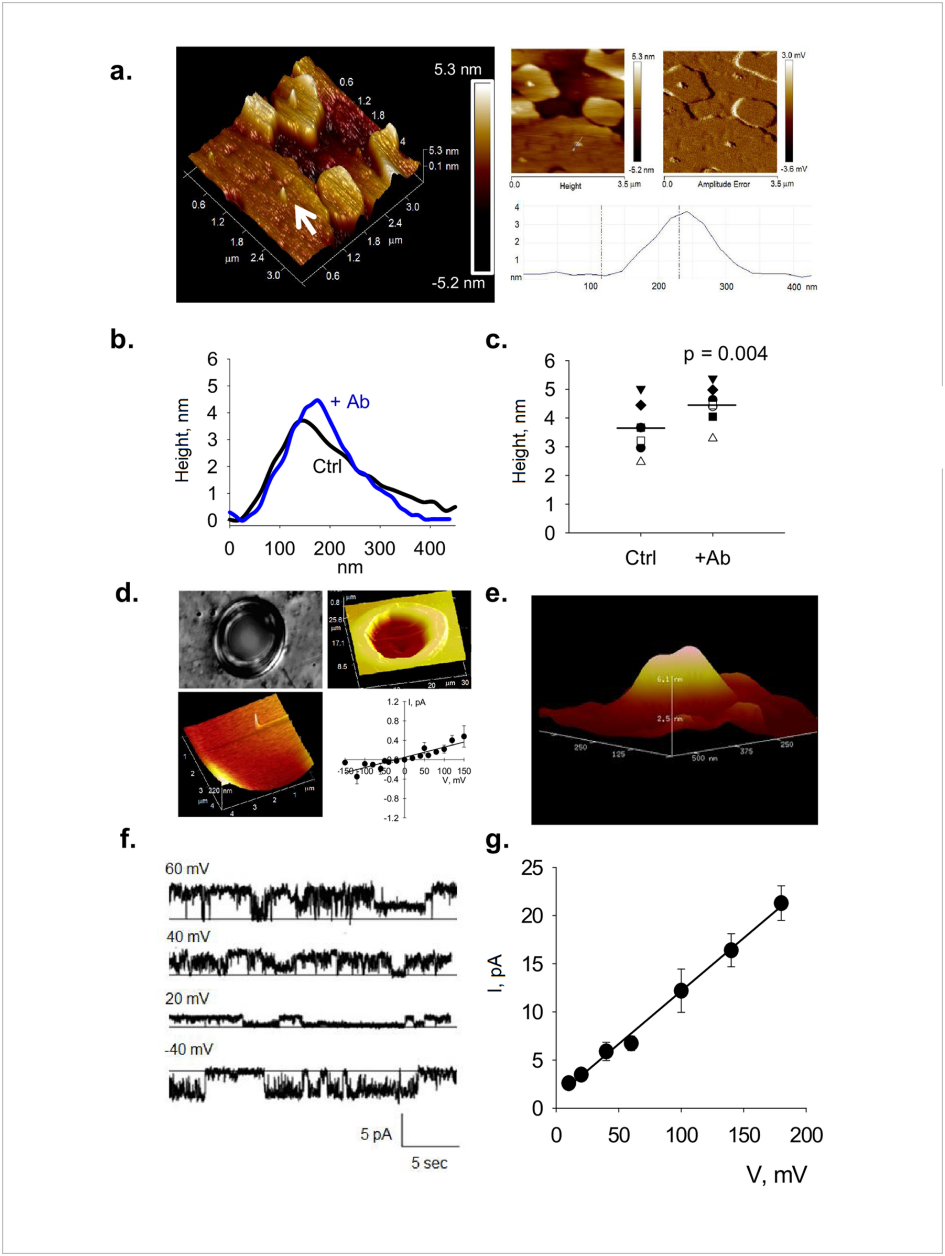
Scheme and data obtained with BLM-AFM microchamber. **a.** AFM imaging of PC2-containing liposomes was conducted on freshly cleaved mica and scanned in saline solution. Left, 3D-perspective of height scan showing the flattened liposomes and the PC2 channel complexes (e.g., white arrow). Right, to better identify the protruding channel complexes on the otherwise clean surface, the height and amplitude error 2D images of material are shown. Bottom Right. AFM height histogram showing the size of a typical channel complex. **b.** Cross section of topological shape of PC2 channels before (Ctrl) and after addition of anti-PC2 antibody (+Ab), which increased the height of the channel. **c.** Height data distributions before (Ctrl), and after addition of anti-PC2 antibody (+Ab). Horizontal lines represent the mean for each condition. **d.** Top. Hole made in the Mylar film shown both as a DIC image (x60, Left), and an AFM scan (Right). Bottom. AFM scan of a clean BLM is shown on the Left, and its current-to-voltage relationship on the Right. **e.** Topological features of a PC2 single channel complex in the BLM. **f.** Single channel tracings of a BLM-imbedded PC2 channel. Holding potentials are shown on top of each recording. Current subconductance states are clearly observed at 60 and 40 mV. **g.** Single channel conductance of the PC2 channel under symmetrical conditions. Data are the mean ± SEM (*n* = 7).

*In vitro* transcription-translation of PC2 was carried out as previously reported [29] with pCI-PKD2 encoding wild-type PC2, using a TNT-T7 coupled reticulocyte lysate system (Promega). The reaction product was diluted with phosphate-buffered saline (400 μl) containing 1% Triton X-100 and 1x protease inhibitor mixture (Roche Diagnostics). PC2-containing proteoliposomes were prepared by addition of *in vitro* translated PC2 diluted 1:1000 in a 7:3 mixture of L-α-phosphatidylcholine (PC, 10 mg/ml, Avanti Polar Lipids, Inc., AL) and 1-palmitoyl-2-oleoyl-Sn-Glycero- 3-phosphatidyl-ethanolamine (PE, 25 mg/ml, Avanti) to get a lipid mixture (40 mg) in n-decane, which was dried with nitrogen gas. The lipid mixture was added 4 ml of Na^+^-cholate buffer (25 mM Na^+^-cholate, 150 mM NaCl, 0.1 mM EDTA, 20 mM HEPES, pH 7.2) and sonicated in a water bath sonicator for 45 min. An aliquot of the diluted PC2-containing solution (1 μl) was added to 100 μl of lipid solution described above, mixed well with 0.2 ml of Na^+^-cholate and dialyzed against dialysis buffer (150 mM NaCl, 0.1 mM EDTA, 20 mM HEPES, pH 7.2) for 3 days at 4°C (with daily changes of buffer). Identification of the channel protein material present in the liposomes was conducted by changes in the topology of the PC2 channel after addition of primary anti-PC2 antibody (200 pM, Catalog number 52–5217 Zymed, South San Francisco, CA, see Fig 2b and 2c), scanned by AFM under conditions reported in [24].

### Electrical recording of PC2 channels

BLMs were formed with a mixture of synthetic phospholipids (Avanti Polar Lipids, Birmingham, AL, USA) in n-decane as previously reported [24, 29]. The lipid mixture contained 1-palmitoyl-2-oleoyl phosphatidyl-choline and phosphatidyl-ethanolamine in a 7:3 ratio. The lipid solution (20–25 mg/ml) was spread over the hole of the Mylar film with a glass capillary. Both sides of the BLM system were bathed with a solution containing 150 mM KCl, and 10 mM Hepes, pH 7.4. Electrical signals were recorded with a PC-501A patch clamp amplifier (Warner Instruments Corp.), using a 10 Gohm feedback resistor. Output signals were low-pass filtered at 1 kHz. Electrical signals were displayed on an oscilloscope (Fig 1a), and digitally stored in a personal computer through a Digidata 1440 A/D converter (Molecular Devices, Sunnyvale, CA). The layout of the entire system, including the electrical connections is shown in Fig 1a. The numbers in parenthesis indicate the actual equipments used, namely (1) ICON AFM, (2) Tecmar Oscilloscope, (3) Digidata 1440 A/D, (4) PC-501A Warner Instruments patch clamp amplifier, and (5) the Nanoscope V controller, respectively. Electrical signals were also fed to an analog input of the AFM controller. Single-channel current tracings were further filtered for display purposes only. Unless otherwise stated, pCLAMP Version 10.2 (Axon Instruments, Foster City, CA, USA) was used for data collection and analysis.

### Atomic force microscopy

PC2 channel complexes were imaged with an ICON AFM attached to a Nano-Scope V controller (Bruker, Sta. Barbara, CA). Unless otherwise stated, samples were scanned with oxide sharpened silicon-nitride tips (DNP-S, Bruker). BLMs were scanned in either tapping or contact mode, in liquid (Fig 1c) with silicon nitride cantilevers with a spring constant of 0.06 N/m and operating frequencies for the tapping mode of 8 kHz (tip radius estimated as ~25 nm). Scanning rates varied from 0.3 to 2 Hz. One line scans in contact mode were conducted at 1–15 Hz, depending on the length of the scanned line (average resolution 100 μs).

### Connection of the patch-clamp amplifier to the AFM

To enable recording of end of frame (EOF) and end of line (EOL) signals, I/O BNC connectors located on the NanoScope V controller were connected to inputs 3 and 4, respectively, of the A/D converter. These connections fed high frequency transistor-transistor logic (TTL) negative voltage pulses (+5 to 0 V) for both signals, such that the electrical signals were back fed into the NanoScope V controller for simultaneous recording of AFM topography and electrophysiology data in the same ASCII file (Fig 1a). To enable this current output, the electrical signal from the patch clamp amplifier was fed into the low frequency BNC input #1 (“low frequency general I/O”). In addition, the output #2 BNC connector under input #1 was connected to both, an input BNC signal connector and the analog #2 input of the Digidata 1440. The connections enabled the visualization of both electrophysiology and height signal scanning data in the same window. The electrophysiology data were saved in same file with the scan ASCII data.

To monitor, record and analyze electrode current output in the AFM, electrical signals were fed into BNC input 1, also referred as “low frequency general I/O”. Output 2 BNC under input 1 was also connected in parallel to both the digital input and #2 analog input of the patch clamp amplifier. These connections enabled the monitoring and analysis of electrical data in the AFM computer in parallel to the AFM scan height window and record electrical data along with the AFM scan data in a single file in ASCII data format.

### Exporting, filtering, and processing AFM scans

AFM scans were exported in ASCII format, and processed entirely offline with NanoScope Analysis 1.5 (Bruker). The number of columns needed were identified, and matched as samples per line, which generated the data that were saved as a text file to be imported into SigmaPlot 11.0 (Systat Software for Windows, Inc). Generation of X and Y column data in ASCII files was conducted as follows. The two-column ASCII file, namely the time and height sensor data, respectively, were added corresponding X axis data from line number, and Y axis data, representing the pixel or sample number in each line. To match electrical data with the scanned image, the requisite portion of the electrical tracing, including the start and end signals of the scan were chosen, converted into an ASCII text file, imported into SigmaPlot and recorded the number of rows (say Y), such that X/Y = N, where if N was an integer, this number was used as a decimation factor to reduce the general I/O height sensor data.

### Statistics

Sigmaplot Version 11.0 (Systat Software for Windows, Inc.) was used for statistical analysis and graphics. Statistical significance was obtained by unpaired Student’s t-test comparison. Data were expressed as the mean ± SEM (*n*), where “*n*” represents the total number of experiments analyzed. Statistical significance was accepted at p < 0.05.

## Results

### Construction and testing of the platform

To initiate a correlation of PC2 single channel tracings with respective topological features of channel structure, we designed and constructed a BLM microchamber platform mounted onto the stage of an AFM that was connected to a conventional patch clamp amplifier as shown in Fig 1a. This setup recorded and correlated simultaneously topological features and electrical currents of single channel complexes. As proof-of-principle of the system, we reconstituted PC2-containing liposomes obtained imbedding *in vitro* translated wild type PC2 into the lipid interface, as previously reported [29, 24]. This procedure entailed the mixing of the protein material with lipids and detergent, followed by extensive dialysis to form and retain PC2-containing liposomes. A similar approach was previously used [24] to extensively characterize the size and topology of the PC2 channel in the lipidic material. Fig 2a shows the material used in the study, where melted PC2-containing liposomes were flattened onto freshly cleaved mica, and identified by AFM scanning. The PC2 channels in the otherwise clean lipidic surface were clearly observed in the scanned images. The PC2 channel complexes were identified by exposure to an anti-PC2 antibody, which increased the height by 0.82 nm (3.63 ± 0.34 nm vs. 4.45 ± 0.25 nm, n = 7, p < 0.004, Fig 2b and 2c). The change in diameter however, did not reach statistical significance. The size and shape of the channel complexes are in agreement with our previously reported data [24], although differences should be expected because the different experimental conditions, including solid support and ionic strength. The BLM support microchamber was assembled from a Nylon spacer glued to a glass microscope slide (Fig 1c), whose top was a Mylar film used to create a BLM supporting flexible interface in which a small clean-edged orifice was created to paint the insulating lipid bilayer. To produce size-defined holes in the Mylar film, orifices were originally carved by AFM etching in contact mode, with silicon cantilevers (Applied NanoStructures, Santa Clara, CA) with a high spring constant of 48 N/m and a resonance frequency of 330 kHz (tip radius estimated as less than 10 nm). These holes were precise in dimension, but rendered unstable BLMs. Thus, a melting approach was developed instead, in which clean-edged round holes were melted in the Mylar film by voltaic arc (Fig 1b, see Materials and methods). Under conditions set for the present study (5 mm distance between the tungsten electrodes), round orifices with an inner diameter in the range of 16.6 ± 2.8 μm (*n* = 8, Fig 2d) were obtained. Once melted by voltaic arc, the Mylar film was stretched and mounted horizontally, such that the orifice was centered and accessible for lipid painting from the upper hemi-chamber. Empty holes were visualized by DIC (x60, Fig 2d, Top Left) and AFM scanning (Fig 2d, Top Right), prior to mounting. Images were taken once again after a BLM was formed in the orifice, with a PC:PE (7:3) lipid mixture in n-decane. The BLM was scanned and imaged by AFM in fluid (Fig 2d, Bottom Left). Both hemi-chambers contained the same KCl solution (150 mM), to collect electrical recordings in the absence of either chemical or osmotic gradients. Once the setup was ready, a seal was formed, as assessed by the large increase in circuit impedance. The BLM conductance was obtained by the electrical response to 500-msec trains of square voltage pulses between ± 200 mV. The resulting, highly linear current-to-voltage relationship was in the order of 2.1 ± 0.3 pS (*n* = 8, Fig 2d, Bottom Right).

### Correlation between single channel currents and topological features of PC2

To determine the electrical properties of PC2 in the microchamber system, the BLM was repainted with PC2-containing liposomes (Fig 2e). Spontaneous single channel currents were observed in more than 365 experiments (Fig 2f). The reconstituted PC2 channels displayed a linear maximal conductance of 110 ± 5 pS (*n* = 3) between ±200 mV in symmetrical KCl (150 mM) (shown here for positive holding potentials only) (Fig 2g). The presence of sub-conductive states was also observed as expected from PC2 channel complexes [29]. The BLM supported continuous scanning over various regions of the entire surface, while obtaining electrical recordings (See Materials and methods). Single channel complexes identified within the scanned BLM (Fig 2e), were with topology similar to that previously reported in flat lipidic environments over mica [24], although consistently taller in the free-floating flexible support under high ionic strength conditions, which is consistent with the expected effect of electrostatic interactions [30].

**Fig 3:**
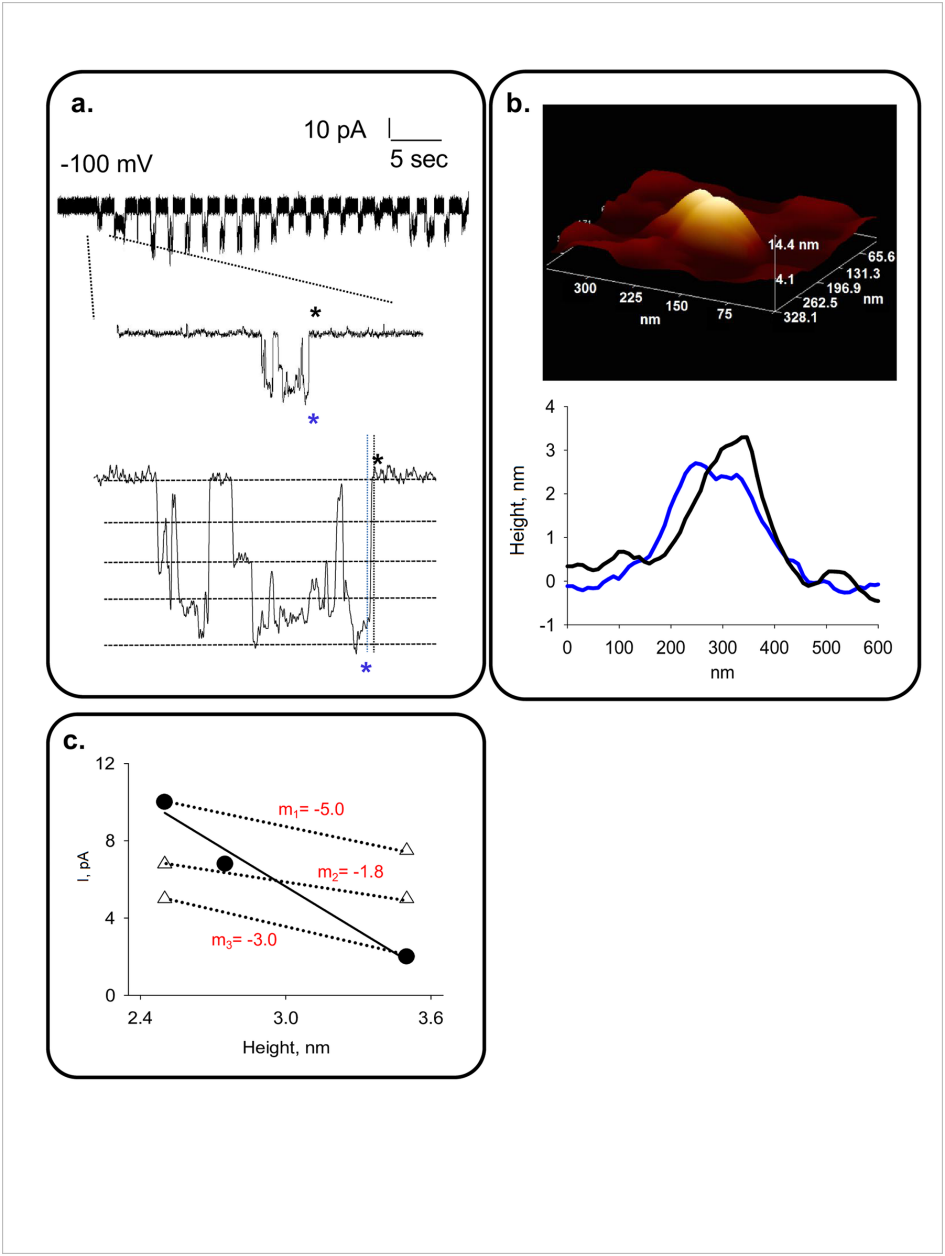
Single channel currents and topological features of PC2. **a.** Top panel, Unfiltered current tracing of a PC2 channel complex activated by AFM scanning over. The single channel opening selected below is further expanded in the Bottom panel. The expanded tracing shows closed open and states, as well as sub-conductance states (dashed lines). **b.** Top panel, AFM scanned 3D image of the channel complexes whose activity is shown in panel **a**. Bottom panel, Single line topological profiles for functional states indicated as Black and Blue asterisks in panel **a**. Please note that proper correlation depends on synchronization of data acquisition systems **c.** Negative linear correlation between height and single channel currents for the channel complex (solid line). A 5 msec shift in synchronization would render different measurements (white triangles), and correlations between conductance states, and thus different negative slopes as indicated (dotted lines). (Data representative of n = 5).

To correlate the PC2 single channel currents with the topological features of identifiable channel complexes, three different approaches were attempted with various degrees of success. In the first approach, AFM scans and patch-clamping electrical recordings were simultaneously collected in their respective systems. Thus, AFM images were collected in the AFM computer, while single channel currents were simultaneously recorded with the BLM system through the A/D converter, and stored in the patch clamp computer. These early findings helped us address the issue of identifying functional single channel complexes in the scanned surface area, because in a few occasions, the AFM tip triggered PC2 channel activity while tapping over the protein complex in the otherwise quiescent BLM (Fig 3a). Unfiltered current tracings of an AFM-tip activated PC2 channel can be observed in Fig 3a, from the scanned complex shown on Fig 3b. Single channel currents and AFM images were matched for the same data collection times (Fig 3c), generating correlated profiles (see Materials and methods) of the respective open and closed channel states from the same single channel complex. Please note that transitions also contained, as expected, conductance substates indicated in dashed lines in Fig 3a (Bottom Panel). The matched data showed an inverse linear correlation between the height of the complex, and the single channel current amplitude, with a slope of 7.63 ± 1.2 pA/nm, *n* = 5, (*r* = 0.9862, Fig 3c). The problem with this approach, however, was the degree of uncertainty in matching the corresponding signals at the microsecond level (nm resolution). Because of the synchronization process an uncertainty time delay could be expected for each trace and retrace scanned line. Thus, current amplitudes within an open burst could have had any one of the values, rendering negative, but very different correlation slopes. Three different slopes, ranging from -1.8 to -5 pA/nm are also indicated in Fig 3c, which can be obtained with a ~5 msec shift in time, based on the presence of substates as shown in Fig 3a.

**Fig 4:**
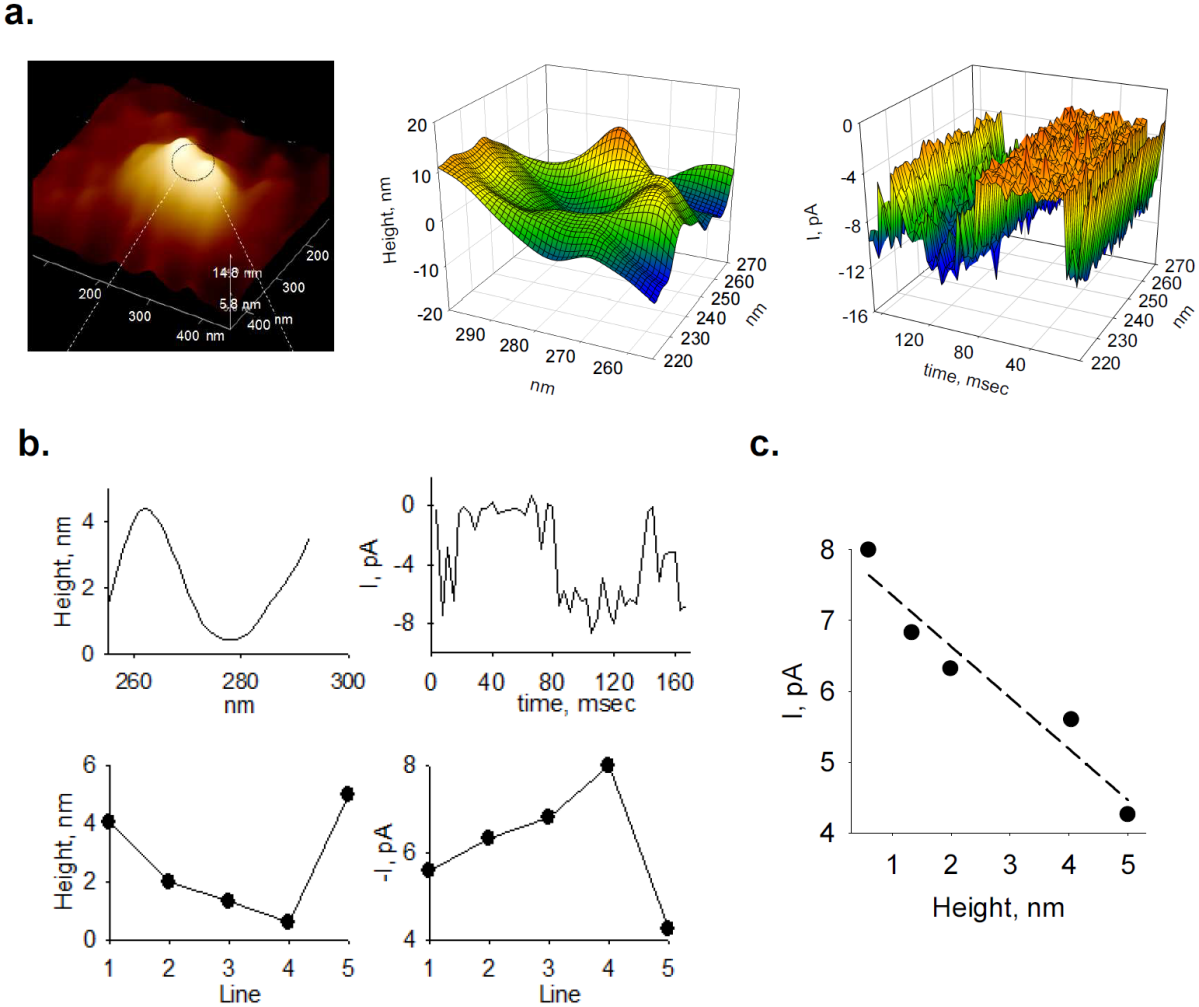
Temporal correlation between PC2 topological features and single channel currents. **a.** (Left) BLM scanned image of a single PC2 channel complex (400 x 400 nm). 3D reconstruction of the channel opening (Middle), and corresponding electrical recordings (Right) from single-line scans as shown in the 3D plot. **b.** Example of single height line of the channel complex (Top Left), and corresponding current amplitude obtained while scanning that line (Top Right). Bottom Panels show corresponding heights and single channel currents for five consecutive single line scans, with an average in-between-lines time of 0.016 msec. **c.** Both sets of experimental data showed an inverse linear correlation for the same channel complex. (Data representative of n = 3).

To improve on this correlation, a second approach was used where the analog electrical currents collected with the BLM electrical system were directly fed into an I/O analog port of the AFM controller (see Materials and methods, and Fig 4). A close up 3D reconstruction (400 x 400 nm) of a single channel complex is shown in Fig 4a, with a “blown up” image of the channel´s mouth in the Middle panel, and its corresponding AFM-collected electrical tracing in the Right panel. An example of the temporal resolution of simultaneous single height line of a channel complex, and its corresponding single channel current during scanning (out of three complexes studied) can be observed in Fig 4b, Upper Panels. Heights and corresponding single channel current amplitudes for five consecutive single line scans, with an average in-between lines time of 0.016 msec, showed an inverse linear correlation (Fig 4b, Bottom Panels) between the two features with slope -0.73 ± 0.31 pA/nm, *n* = 3 (*r* = 0.9642, Fig 4c). It is important to note that when both signals were registered in the AFM system, the uncertainty of matching scanning lines and single channel currents was reduced, but was not eliminated, as AFM trace-retraced scanned lines contained coding instructions that had to be eliminated by hand after each run (see Materials and methods).

**Fig 5:**
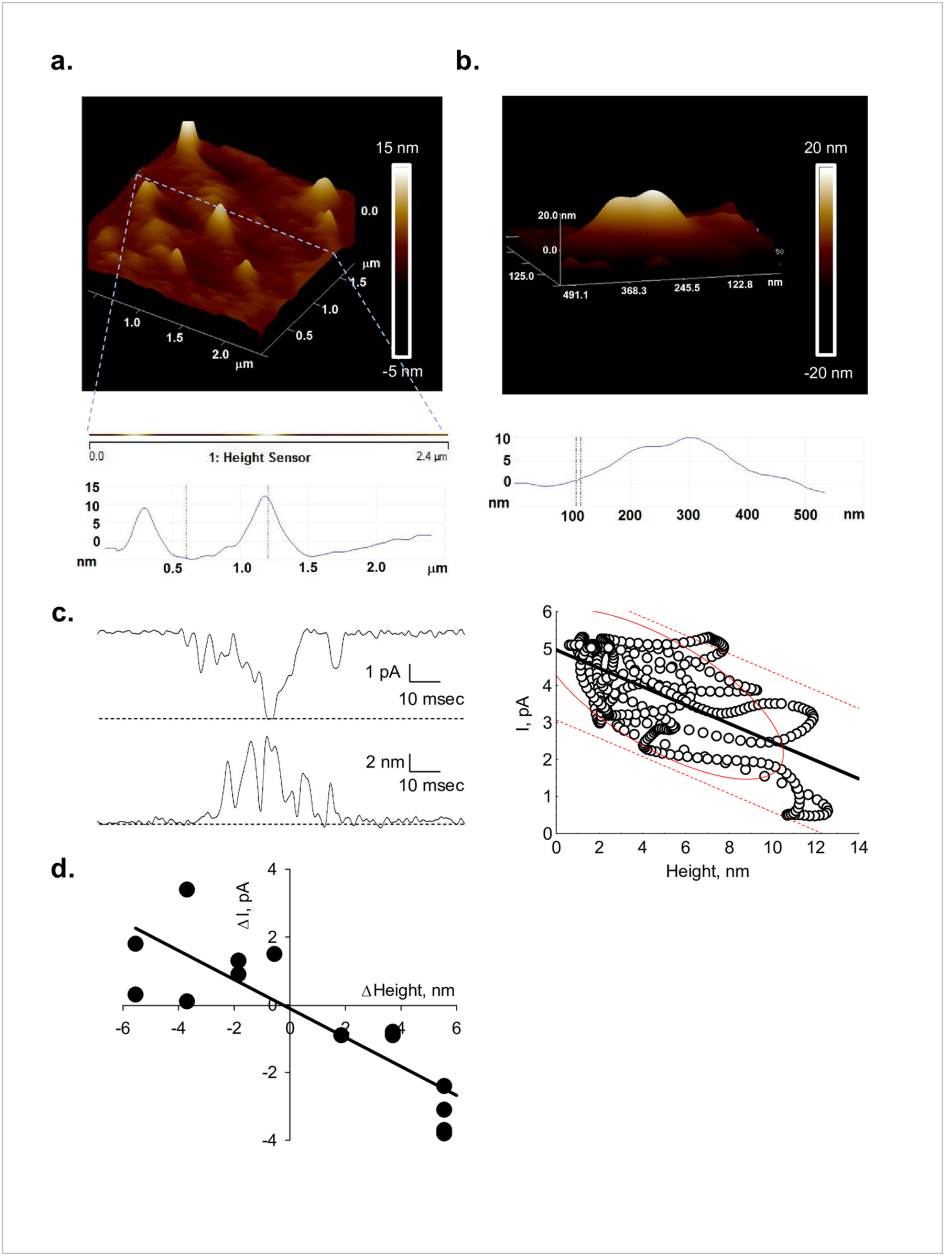
Electrical and topological correlates from single line scans of PC2. **a.** (Top) Large BLM scan (2.4 x 2.4 μm) used to locate single channel complexes (white peaks). Dashed line indicates position of fast one-line scanning. (Middle) Enlarged two-line scan to show location of channel complexes in the image. (Bottom) Height profiles of two channel complexes traversed by the scan line. **b.** Smaller scan (500 x 500 nm) showing an identified single channel complex and corresponding height profile (below). **c.** Left**.** Short single channel current tracing (110 msec) showing sub-conductance levels (Top) and corresponding height profile obtained from simultaneous single-line scans (Bottom). Right. Correlation between height and current amplitude (dashed red lines indicate data ellipse and regression bands with 95% confidence interval). **d.** Changes in relative height and current amplitude respect to the longer dwell time for each scan (see text), obtained for another channel complex again showing an inverse linear correlation.

To further improve on the matching times for data acquisition from both setups, PC2 single channel currents and AFM heights were simultaneously collected with the patch clamping system instead. The output signal from the AFM machine was fed into the A/D converter interfacing the electrical acquisition system (Fig 5). Thus, data were collected both as analog signals and analyzed directly with the Clampfit 10.2 software, and comparisons were then readily made between the AFM scan lines and the BLM electrical data. For these experiments, large BLM areas (e.g. 2.4 x 2.4 μm) were first scanned to locate single channel complexes (white peaks, Fig 5a). Once identified, height profiles were obtained showing single-line scans (Fig 5a, Middle panel) over the channel complex. Smaller surface areas (e.g. 500 x 500 nm scans) were also scanned over the identified channel complexes to determine topological features and height profiles (Fig 5b). Fast one-line scans (15 Hz) of the channel complex could then be obtained, and matched to corresponding single channel tracings, identifying single openings and closing events (Fig 5c). This method reduced scanning time regions without ion channels. The correlation between changes in channel´s height and the ionic currents (open trace on top), is shown as a series of point-by-point height-to-current coordinates showing an inverse linear correlation with a slope of -0.25 ± 0.02 pA/nm (*r* = -0.6582). Changes in height (namely positive and negative deflections for closing and opening states, respectively) from another channel complex are also shown, with similar results (slope of -0.43 ± 0.07 pA/nm, *r* = 0.8690, Fig 5d).

## Discussion

The present study describes the construction and practical use of a BLM platform that allows simultaneous collection of electrical data and AFM topological features from single channel complexes. Proof-of-principle was successfully achieved with the TRP channel polycystin-2. This approach departs from other traditional methods in that it provides both structural and functional information from “live” single channel complexes. Traditionally, ion channel behavior is functionally characterized by electrophysiological techniques such as patch-clamping and BLM reconstitution that rendered real-time electrical information from single molecular complexes. In contrast, no method is yet available to gather topological features from live single channel molecules [3, 4]. Structural information of channel complexes requires independent techniques such as cryo-EM that render high resolution topological information, but require a number of averaging methods of multiple copies to achieve high resolution. No structural information is yet available that is directly obtained from single molecules. Although not as powerful as cryo-EM, AFM is also a very powerful imaging technique that provides topological information from functional molecular complexes and the advantage that data acquisition can be carried out under physiological conditions in native aqueous environments [23, 24]. We previously used AFM imaging to determine the tetrameric topology and dimensions, including height and size of the protruding volume of single channel complexes of both PC2 and TRPC1, as well as hetero-multimeric complexes thereof. Data in that study were obtained in flattened liposomes on mica, in aqueous solution under low ionic strength conditions [24]. Although we also reported single channel currents from identical samples, no direct structural-functional correlations of the channel complexes were attempted, as both techniques were conducted independently. No insights into structural-functional correlates from live channels are yet available in the literature, despite the fact that similar studies have gathered single channel BLM electrophysiological data and AFM imaging from identical samples [26, 31].

The main issue concerning simultaneous data acquisition from both techniques lies in the fact that conventional BLM chambers have very thick supporting walls, where ion channel-containing BLMs are actually inaccessible to the AFM tip. This drawback could be solved if a sufficiently thin material (< 10 μm) is used to construct the supporting wall that holds the BLM. Previous attempts in this regard, attained variable success. Ovalle-García & Ortega-Blake [32] for example, combined AFM and the patch-clamping technique to study ion channel properties in BLMs, where a Mylar film was carved with an AFM-driven glass microneedle. A BLM was painted in the hole that was doped with gramicidin D channels that were observed and tested by AFM scanning. The study did not collect simultaneous electrical and topological information on the same channel complexes. Lal’s group also described a silicon chip-based supported bilayer system (Lab-on-a-chip) [31] to detect the presence of ion channels and their electrical conductance in BLMs. Nanopores were produced in microfabricated silicon membranes, where the AFM tip was actually used as an electrode to measure gramicidin-mediated electrical currents. Again, the study did not report any simultaneous electrical records and AFM topological features from the functional channel complexes.

In this study, we constructed a horizontal BLM microchamber, where the actual interface was a hole-bearing thin film of Mylar from which single channel complexes could be imbedded and studied both electrically and topologically. One of the advantages of this microsystem was that both the size and intrinsic resistance of the BLM were similar to those of isolated membrane patches (> 1 GΩ). The main advantage of the present setup, however, was the fact that, while collecting single channel electrical currents, the BLM surface could also be readily scanned by AFM, such that the imbedded ion channels were identified and imaged. We reconstituted liposomes containing *in vitro* translated PC2, a TRP-type cation channel we have extensively studied in the past [24, 29]. In agreement with our previous reports, the reconstituted PC2 channels could be functionally characterized, displaying their characteristic large single channel conductance and paradigmatic sub-conductance states [24, 29]. To unequivocally ascribe the topological changes in the scans to the PC2 channels inserted in the BLM and not any other potential contaminants. We tested the effect of an anti-PC2 antibody, which indeed correlated with an increase in the height of the PC2 channel, as previously reported [29].

AFM scanning generally overestimates the diameter of biological samples due to the geometry of the tip, which induces image broadening effects [33]. In fact, AFM imaging of ion channels in saline solution renders a calculated volume that although more physiological in nature, it does not easily correlate with other methods, because it includes the partial specific volume of the protein, a volume associated with the extent of protein hydration, and possibly structural lipids associated with the channel complex itself. We previously reported the “size” of the PC2 channel complex in lipid membranes [24] obtained from cross-sections of individual protein complexes by measuring the mean height and diameter of each single particle. Following Schneider et al, [33], the calculated PC2 volume from the AFM dimensions was 8.695 nm^3^, for the “tall” tetramer [24], which was 16 times larger than the expected volume obtained from its amino acid sequence (~135 nm^3^ / PC2 monomer, or 540 nm^3^ / tetramer, as calculated from method reported in [34]). Interestingly, the data were in agreement with reported information obtained by cryo-EM of other TRP channels. Maruyama et al., for example, reported a volume of 8.341 nm^3^ for the cryo-EM 3D reconstruction of TRPM2, which was 9.61 times larger than its theoretical volume of 867.6 nm^3^ [28]. Similarly, Mio et al. [27] reported an approximate hydrated volume of 7.379 nm^3^ for TRPC3, which is 15.2 times larger than its theoretical volume (486 nm^3^). Differences between AFM dimensions and theoretical volumes could also be explained by conformational changes expected in the transmembrane structure of the channel protein in a lipid bilayer, and also by electrostatic interactions [31]. Thus, differences in AFM imaging will be expected based on the support in which it is made, for example, mica or lipid surfaces. An AFM image of the syncollin monomer, for example, rendered a molecular volume similar to its theoretical value as a naked protein on mica [35]. After insertion of the protein into a lipid bilayer however, syncollin showed a doughnut shape consistent with a hexamer, rendering a monomer volume four times larger than expected. The apparent height of the PC2 channel complex in the free-floating BLM was consistently larger than those observed in mica. Nonetheless, changes in height associated with functional states were always self-referred and thus, relevant to the structural-functional correlates of the live channels.

The location of single ion channel complexes in the surface area of the sealed BLM was preliminarily conducted in large scans, which did not modify the conductance of the BLM. Our ability to search and choose smaller scanning areas within the BLM allowed us to simultaneously record of single channel currents and topological features from identifiable single channel complexes. The combined experimental data provided evidence for an inverse correlation between peak single channel currents and the overall height of the PC2 channel complex. Because the PC2 channel has intrinsic subconductance states associated with the monomeric contributions in the channel complex, our findings are consistent with the possibility that the transmembrane domains of the channel that define the conductance pore protrude more or less from the entire structure, as single channel currents decrease to the closed state and vice versa.

Presently, no experimental evidence is available as to the correlation between the functional state of an ion channel and its height. However, a recent report by Sumino et al used an integrated single-molecule approach that included AFM, molecular dynamics (MD) simulation, and single-channel recordings, to recreate a picture of both the dynamic structure and function of the activation gate of the KcsA K^+^ channel [36]. This combined model indicated that KcsA shortens its longitudinal length upon opening. An interesting finding in the Sumino study was, however, the fact that both AFM and crystal structure only rendered a similar height for the closed conformation, while showing substantial discrepancies between measurements in the open conformation. The crystallographically unsolved carboxy-terminal residues involving the H_bulge_, extended towards the cytoplasmic space, and only AFM imaging was able to reveal that the cytoplasmic end of the transmembrane pore domain actually protruded from the membrane surface such that the longitudinal length of the channel was substantially shortened upon channel opening. The AFM images rendered a clear distinction between the closed and open gate structures [36].

To date comprehensive structural experimental evidence of the transitions between conformational open and closed states of ion channel is largely unavailable. TRP channels share topological similarities with voltage-gated K^+^ channels that sense the imposed electric field through four peripheral voltage sensing domains around a single ion conducting pore [37], which undergo conformational changes upon membrane depolarization. It is currently accepted that upon depolarization, the positively charged arginine-rich S4 helix, are driven toward the outside, thus eliciting the pore opening. A number of studies, including pioneering work by MacKinnon´s group have reported on the expected changes in voltage-sensitive K^+^ channel structures, including a 20Å shift between the open and closed states [38], which is largely similar to the displacements observed in the present study. Thus, the method reported herein provides comparable evidence, with the advantage of obtaining simultaneous correlations with the electrical properties of live single ion channels.

Another novel finding emanating from the simultaneous topological and electrical measurements of identified PC2 channels was the mechanical response to the scanning tip. In a few instances (*n* = 5), single channel currents were induced and controlled by the actual force imposed on the channel by the AFM tip (spring constant 0.06 N/m), either over, or close to the channel complex (Fig 3a). The mechanical force of the responsive channels however, was consistent with activation energy in the order of 0.04 pJ for channel gating, thus suggesting an intrinsic mechanosensitivity of PC2. Because BLM imbedded channels were not consistently activated by AFM scanning of the entire surface area, and the ones mechanically responsive were only a small fraction of the scanned channels, it is possible that sidedness of the reconstituted channel may also play a role in this activation process, a phenomenon that will also require further investigation.

In summary, we report on the design and successful application of an experimental platform for the simultaneous acquisition of topological features of “live” single ion channels by AFM imaging, while acquiring electrical information from the same channel complex. The evidence disclosed an inverse correlation between the magnitude of the PC2 single channel conductance and its height in the BLM. This combined experimental approach also provided support for the intrinsic mechanosensitivity of PC2. The present method opens up the possibility to explore direct high resolution structural-functional correlations from single “live” molecules in their native environments. Future advances with improved technologies such as temporal resolution with high-speed AFM imaging [39] will further expand on direct comparisons at higher rates and longer, continuous tracings of functional channels.
